# (Anthracen-9-ylmeth­yl)benzyl­ammonium chloride

**DOI:** 10.1107/S1600536811016837

**Published:** 2011-05-20

**Authors:** Jian-Rong Han, Wei Wang, Xiao-Li Zhen, Xia Tian, Shou-Xin Liu

**Affiliations:** a College of Sciences, Hebei University of Science & Technology, Shijiazhuang 050018, People’s Republic of China; b College of Chemical & Pharmaceutical Engineering, Hebei University of Science & Technology, Shijiazhuang 050018, People’s Republic of China

## Abstract

In the title compound, C_22_H_20_N^+^·Cl^−^, the anthracene system makes a dihedral angle of 72.65 (4)° with the benzene ring. The C—N—C—C torsion angles in the chain connecting the benzene ring and anthracene system are 52.24 (15) and −170.73 (11)°. The crystal structure is stabilized by inter­molecular N—H⋯Cl and C—H⋯Cl hydrogen bonds, which link the mol­ecules into tetra­mers about inversion centers.

## Related literature

For the synthesis and structures of related compounds, see: Ashton *et al.* (1997 ▶). For formation of rotaxanes from *sec*-ammonium salts and crown ethers, see: Nakazono *et al.* (2008 ▶).
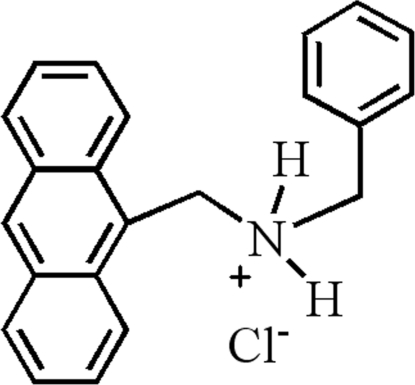


## Experimental

### 

#### Crystal data

C_22_H_20_N^+^·Cl^−^
*M* *_r_* = 333.84
Triclinic, 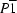

*a* = 6.7457 (13) Å
*b* = 10.761 (2) Å
*c* = 13.033 (3) Å
α = 94.45 (3)°
β = 104.84 (3)°
γ = 104.48 (3)°
*V* = 875.3 (3) Å^3^
*Z* = 2
Mo *K*α radiation
μ = 0.22 mm^−1^
*T* = 113 K
0.28 × 0.24 × 0.20 mm

#### Data collection

Rigaku Saturn CCD area-detector diffractometer
Absorption correction: multi-scan (*CrystalClear*; Rigaku/MSC, 2005 ▶) *T*_min_ = 0.941, *T*_max_ = 0.957
5882 measured reflections
3048 independent reflections
2342 reflections with *I* > 2σ(*I*)
*R*_int_ = 0.027

#### Refinement

*R*[*F* ^2^ > 2σ(*F* ^2^)] = 0.031
*wR*(*F* ^2^) = 0.088
*S* = 1.04
3048 reflections
226 parameters
3 restraints
H atoms treated by a mixture of independent and constrained refinement
Δρ_max_ = 0.20 e Å^−3^
Δρ_min_ = −0.21 e Å^−3^

### 

Data collection: *CrystalClear* (Rigaku/MSC, 2005 ▶); cell refinement: *CrystalClear*; data reduction: *CrystalClear*; program(s) used to solve structure: *SHELXS97* (Sheldrick, 2008 ▶); program(s) used to refine structure: *SHELXL97* (Sheldrick, 2008 ▶); molecular graphics: *SHELXTL* (Sheldrick, 2008 ▶); software used to prepare material for publication: *SHELXTL*.

## Supplementary Material

Crystal structure: contains datablocks I, global. DOI: 10.1107/S1600536811016837/pv2409sup1.cif

Structure factors: contains datablocks I. DOI: 10.1107/S1600536811016837/pv2409Isup2.hkl

Supplementary material file. DOI: 10.1107/S1600536811016837/pv2409Isup3.cml

Additional supplementary materials:  crystallographic information; 3D view; checkCIF report

## Figures and Tables

**Table 1:** Hydrogen-bond geometry (Å, °)

*D*—H⋯*A*	*D*—H	H⋯*A*	*D*⋯*A*	*D*—H⋯*A*
N1—H1*A*⋯Cl1^i^	0.92 (1)	2.26 (1)	3.0963 (13)	152 (1)
N1—H1*B*⋯Cl1	0.92 (1)	2.17 (1)	3.0781 (16)	170 (1)
C16—H16*A*⋯Cl1^ii^	0.97	2.60	3.4824 (16)	151

Symmetry codes: (i) 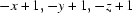; (ii) 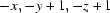.

**Fig. 1.**
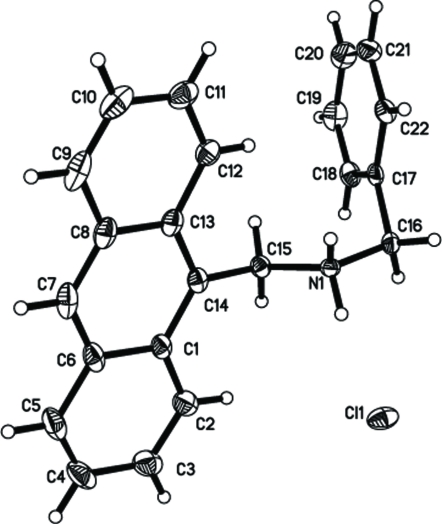
The molecular structure of the title complex, with 50% probablity ellipsoids.

**Fig. 2.**
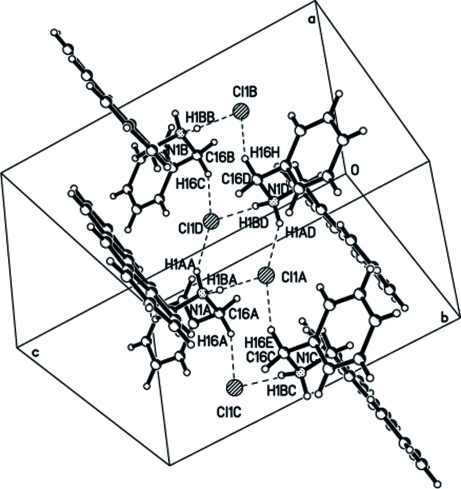
Unit cell packing of the title complex, showing hydrogen bonded tetramers.

## supplementary crystallographic information

### Comment

Sec-ammonium salts and crown ethers combine well to yield a stable pseudorotaxanes as a precursor of rotaxanes (Nakazono *et al.*, 2008). (9-Anthracenyl) benzylammonium hexafluorophosphate and aromatic crown ethers give hydrogen-bonded complexes pseudorotaxane-like geometries (Ashton *et al.*, 1997). In this paper we report the synthesis and crystal strucure of (9-anthracenyl) benzyl ammonium chloride.

In the title compound (Fig. 1), anthracene ring makes a dihedral angle of 72.65 (4)° with benzene ring. The torsion angles in the chain connecting the benzene and the anthracene rings, (C15/N1—C16/C17 and (C16/N1—C15/C14) are 52.24 (15) ° and -170.73 (11)°, respectively. In the crystal structure, the crystal packing is stabilized by intermolecular N1—H1A···Cl1 and C16—H16A···Cl1 hydrogen bonds which link the molecules into tetramers about inversion centers (Table 1 and Fig. 2).

### Experimental

A mixture of 9-anthracenealdehyde (6.18 g, 30 mmol) and benzylamine (3.86 g, 36 mmol) and molecular Sieve in toluene (200 mL) was heated under reflux with stirring in a water divider for 10 h. After the reaction mixture had cooled down to room temperature, the solvent was removed in vacuo to give the imine. The solid was dissolved in hot MeOH (150 mL), followed by drop-wise addition of NaBH_4_ (5.70 g, 150 mmol) and heating under reflux with stirring for 8 h. The reaction mixture was then allowed to cool down to room temperature, and concentrated HCl was added (pH<2). After evaporation of the solvent, the residue was suspended in H_2_O (70 mL) and extracted with CH_2_Cl_2_ (4 × 50 mL). The combined extracts were washed with 5% aqueous NaHCO_3_ (2× 70 mL) and H_2_O (70 mL) and then dried (MgSO_4_). Removal of the solvent in vacuo afforded the (9-anthracenyl)benzyl amine which was treated according to literature (Ashton *et al.*, 1997) to prepare the title compound. Pale yellow single crystals of the title compound suitable for X-ray analysis were obtained by slow evaporation of an methnol solution.

### Refinement

The H atoms were included at calculated positions with C—H = 0.93 and 0.97 Å for aryl and methylene type H-atoms, respectively, and refined in a riding mode with *U*_iso_(H) = 1.2*U*_eq_(C). The amino H-atoms were located from a difference Fourier map and were allowed to refine freely.

### Crystal data

**Table:**

C_22_H_20_N^+^·Cl^−^	*Z* = 2
*M**_r_* = 333.84	*F*(000) = 352
Triclinic, *P*1	*D*_x_ = 1.267 Mg m^−^^3^
Hall symbol: -P 1	Mo *K*α radiation, λ = 0.71073 Å
*a* = 6.7457 (13) Å	Cell parameters from 2756 reflections
*b* = 10.761 (2) Å	θ = 2.4–27.9°
*c* = 13.033 (3) Å	µ = 0.22 mm^−^^1^
α = 94.45 (3)°	*T* = 113 K
β = 104.84 (3)°	Block, yellow
γ = 104.48 (3)°	0.28 × 0.24 × 0.20 mm
*V* = 875.3 (3) Å^3^	

### Data collection

**Table:**

Rigaku Saturn CCD area-detector diffractometer	3048 independent reflections
Radiation source: rotating anode	2342 reflections with *I* > 2σ(*I*)
confocal	*R*_int_ = 0.027
Detector resolution: 7.31 pixels mm^-1^	θ_max_ = 25.0°, θ_min_ = 2.4°
ω and φ scans	*h* = −8→8
Absorption correction: multi-scan (*CrystalClear*; Rigaku/MSC, 2005)	*k* = −12→10
*T*_min_ = 0.941, *T*_max_ = 0.957	*l* = −15→15
5882 measured reflections	

### Refinement

**Table:**

Refinement on *F*^2^	Secondary atom site location: difference Fourier map
Least-squares matrix: full	Hydrogen site location: inferred from neighbouring sites
*R*[*F*^2^ > 2σ(*F*^2^)] = 0.031	H atoms treated by a mixture of independent and constrained refinement
*wR*(*F*^2^) = 0.088	*w* = 1/[σ^2^(*F*_o_^2^) + (0.0502*P*)^2^] where *P* = (*F*_o_^2^ + 2*F*_c_^2^)/3
*S* = 1.04	(Δ/σ)_max_ = 0.002
3048 reflections	Δρ_max_ = 0.20 e Å^−^^3^
226 parameters	Δρ_min_ = −0.21 e Å^−^^3^
3 restraints	Extinction correction: *SHELXL97* (Sheldrick, 2008), Fc^*^=kFc[1+0.001xFc^2^λ^3^/sin(2θ)]^-1/4^
Primary atom site location: structure-invariant direct methods	Extinction coefficient: 0.152 (9)

### Special details

**Table:**

Geometry. All e.s.d.'s (except the e.s.d. in the dihedral angle between two l.s. planes) are estimated using the full covariance matrix. The cell e.s.d.'s are taken into account individually in the estimation of e.s.d.'s in distances, angles and torsion angles; correlations between e.s.d.'s in cell parameters are only used when they are defined by crystal symmetry. An approximate (isotropic) treatment of cell e.s.d.'s is used for estimating e.s.d.'s involving l.s. planes.
Refinement. Refinement of *F*^2^ against ALL reflections. The weighted *R*-factor *wR* and goodness of fit *S* are based on *F*^2^, conventional *R*-factors *R* are based on *F*, with *F* set to zero for negative *F*^2^. The threshold expression of *F*^2^ > σ(*F*^2^) is used only for calculating *R*-factors(gt) *etc*. and is not relevant to the choice of reflections for refinement. *R*-factors based on *F*^2^ are statistically about twice as large as those based on *F*, and *R*- factors based on ALL data will be even larger.

### Fractional atomic coordinates and isotropic or equivalent isotropic displacement parameters (Å^2^)

**Table:**

	*x*	*y*	*z*	*U*_iso_*/*U*_eq_	
Cl1	0.17351 (5)	0.42040 (4)	0.40032 (3)	0.02288 (15)	
N1	0.38238 (18)	0.52936 (11)	0.64009 (9)	0.0139 (3)	
C1	0.1631 (2)	0.21072 (14)	0.67206 (11)	0.0184 (3)	
C2	−0.0472 (2)	0.21057 (16)	0.61273 (12)	0.0242 (4)	
H2	−0.0774	0.2890	0.6018	0.029*	
C3	−0.2034 (3)	0.09727 (16)	0.57217 (13)	0.0305 (4)	
H3	−0.3391	0.0995	0.5340	0.037*	
C4	−0.1629 (3)	−0.02440 (17)	0.58713 (13)	0.0341 (4)	
H4	−0.2721	−0.1009	0.5597	0.041*	
C5	0.0344 (3)	−0.02903 (16)	0.64127 (12)	0.0305 (4)	
H5	0.0595	−0.1091	0.6505	0.037*	
C6	0.2045 (3)	0.08678 (15)	0.68441 (12)	0.0227 (4)	
C7	0.4101 (3)	0.08322 (16)	0.73768 (12)	0.0262 (4)	
H7	0.4365	0.0029	0.7438	0.031*	
C8	0.5764 (2)	0.19449 (15)	0.78173 (11)	0.0223 (4)	
C9	0.7883 (3)	0.18991 (18)	0.83401 (12)	0.0299 (4)	
H9	0.8163	0.1098	0.8382	0.036*	
C10	0.9485 (3)	0.29980 (18)	0.87736 (12)	0.0315 (4)	
H10	1.0853	0.2948	0.9103	0.038*	
C11	0.9084 (2)	0.42185 (17)	0.87262 (11)	0.0273 (4)	
H11	1.0193	0.4969	0.9030	0.033*	
C12	0.7099 (2)	0.43137 (16)	0.82427 (11)	0.0221 (4)	
H12	0.6871	0.5130	0.8231	0.026*	
C13	0.5357 (2)	0.31906 (15)	0.77525 (11)	0.0188 (3)	
C14	0.3287 (2)	0.32511 (14)	0.72005 (11)	0.0170 (3)	
C15	0.2884 (2)	0.45631 (14)	0.71774 (11)	0.0167 (3)	
H15A	0.1356	0.4454	0.6977	0.020*	
H15B	0.3499	0.5065	0.7891	0.020*	
C16	0.3787 (2)	0.66897 (14)	0.64641 (11)	0.0159 (3)	
H16A	0.2322	0.6725	0.6209	0.019*	
H16B	0.4571	0.7116	0.6000	0.019*	
C17	0.4765 (2)	0.74042 (14)	0.75999 (11)	0.0160 (3)	
C18	0.3455 (2)	0.75556 (14)	0.82421 (12)	0.0214 (4)	
H18	0.1978	0.7247	0.7963	0.026*	
C19	0.4352 (3)	0.81680 (15)	0.93001 (12)	0.0277 (4)	
H19	0.3472	0.8261	0.9729	0.033*	
C20	0.6531 (3)	0.86365 (16)	0.97157 (13)	0.0300 (4)	
H20	0.7122	0.9046	1.0424	0.036*	
C21	0.7851 (3)	0.84988 (15)	0.90801 (12)	0.0254 (4)	
H21	0.9326	0.8817	0.9360	0.030*	
C22	0.6962 (2)	0.78841 (14)	0.80265 (11)	0.0194 (3)	
H22	0.7848	0.7793	0.7601	0.023*	
H1A	0.5219 (14)	0.5298 (15)	0.6487 (11)	0.027 (4)*	
H1B	0.3065 (17)	0.4908 (14)	0.5707 (8)	0.018 (4)*	

### Atomic displacement parameters (Å^2^)

**Table:**

	*U*^11^	*U*^22^	*U*^33^	*U*^12^	*U*^13^	*U*^23^
Cl1	0.0145 (2)	0.0350 (3)	0.0170 (2)	0.00489 (17)	0.00408 (14)	−0.00094 (16)
N1	0.0128 (6)	0.0149 (7)	0.0127 (6)	0.0023 (5)	0.0032 (5)	0.0016 (5)
C1	0.0249 (8)	0.0171 (8)	0.0153 (7)	0.0035 (7)	0.0110 (6)	0.0039 (6)
C2	0.0265 (9)	0.0237 (9)	0.0219 (8)	0.0031 (7)	0.0102 (7)	0.0024 (7)
C3	0.0249 (9)	0.0339 (11)	0.0270 (9)	−0.0032 (8)	0.0090 (7)	0.0034 (8)
C4	0.0413 (11)	0.0230 (9)	0.0302 (9)	−0.0108 (8)	0.0172 (8)	0.0002 (8)
C5	0.0479 (11)	0.0167 (9)	0.0285 (9)	0.0017 (8)	0.0208 (8)	0.0045 (7)
C6	0.0365 (10)	0.0161 (8)	0.0194 (8)	0.0044 (7)	0.0171 (7)	0.0039 (7)
C7	0.0434 (10)	0.0212 (9)	0.0259 (8)	0.0176 (8)	0.0201 (8)	0.0109 (7)
C8	0.0321 (9)	0.0257 (9)	0.0171 (7)	0.0140 (8)	0.0138 (7)	0.0085 (7)
C9	0.0413 (10)	0.0403 (11)	0.0247 (9)	0.0288 (9)	0.0174 (8)	0.0174 (8)
C10	0.0261 (9)	0.0532 (12)	0.0221 (8)	0.0187 (9)	0.0089 (7)	0.0148 (8)
C11	0.0264 (9)	0.0391 (10)	0.0164 (8)	0.0077 (8)	0.0065 (7)	0.0068 (7)
C12	0.0253 (8)	0.0254 (9)	0.0164 (7)	0.0073 (7)	0.0069 (6)	0.0048 (7)
C13	0.0251 (8)	0.0225 (8)	0.0138 (7)	0.0095 (7)	0.0107 (6)	0.0056 (6)
C14	0.0226 (8)	0.0167 (8)	0.0157 (7)	0.0061 (6)	0.0111 (6)	0.0039 (6)
C15	0.0176 (8)	0.0164 (8)	0.0173 (7)	0.0041 (6)	0.0075 (6)	0.0033 (6)
C16	0.0159 (7)	0.0143 (8)	0.0189 (7)	0.0049 (6)	0.0056 (6)	0.0059 (6)
C17	0.0197 (8)	0.0097 (7)	0.0197 (7)	0.0051 (6)	0.0056 (6)	0.0053 (6)
C18	0.0231 (8)	0.0166 (8)	0.0277 (8)	0.0078 (7)	0.0099 (7)	0.0057 (7)
C19	0.0395 (10)	0.0256 (9)	0.0255 (8)	0.0147 (8)	0.0168 (7)	0.0038 (7)
C20	0.0439 (10)	0.0219 (9)	0.0211 (8)	0.0095 (8)	0.0049 (7)	−0.0015 (7)
C21	0.0251 (8)	0.0180 (8)	0.0269 (8)	0.0029 (7)	0.0006 (7)	0.0008 (7)
C22	0.0216 (8)	0.0148 (8)	0.0224 (8)	0.0046 (6)	0.0079 (6)	0.0031 (6)

### Geometric parameters (Å, °)

**Table:**

N1—C15	1.4990 (17)	C10—H10	0.9300
N1—C16	1.5051 (18)	C11—C12	1.359 (2)
N1—H1A	0.917 (8)	C11—H11	0.9300
N1—H1B	0.922 (8)	C12—C13	1.426 (2)
C1—C14	1.410 (2)	C12—H12	0.9300
C1—C2	1.431 (2)	C13—C14	1.418 (2)
C1—C6	1.442 (2)	C14—C15	1.504 (2)
C2—C3	1.360 (2)	C15—H15A	0.9700
C2—H2	0.9300	C15—H15B	0.9700
C3—C4	1.421 (2)	C16—C17	1.512 (2)
C3—H3	0.9300	C16—H16A	0.9700
C4—C5	1.352 (2)	C16—H16B	0.9700
C4—H4	0.9300	C17—C22	1.386 (2)
C5—C6	1.425 (2)	C17—C18	1.3922 (19)
C5—H5	0.9300	C18—C19	1.391 (2)
C6—C7	1.394 (2)	C18—H18	0.9300
C7—C8	1.383 (2)	C19—C20	1.374 (2)
C7—H7	0.9300	C19—H19	0.9300
C8—C9	1.432 (2)	C20—C21	1.388 (2)
C8—C13	1.438 (2)	C20—H20	0.9300
C9—C10	1.353 (2)	C21—C22	1.386 (2)
C9—H9	0.9300	C21—H21	0.9300
C10—C11	1.408 (2)	C22—H22	0.9300
			
C15—N1—C16	114.67 (10)	C11—C12—C13	121.55 (16)
C15—N1—H1A	112.3 (10)	C11—C12—H12	119.2
C16—N1—H1A	106.5 (10)	C13—C12—H12	119.2
C15—N1—H1B	109.8 (9)	C14—C13—C12	123.26 (14)
C16—N1—H1B	106.0 (9)	C14—C13—C8	119.31 (14)
H1A—N1—H1B	107.2 (9)	C12—C13—C8	117.42 (14)
C14—C1—C2	123.40 (14)	C1—C14—C13	120.77 (14)
C14—C1—C6	118.93 (14)	C1—C14—C15	120.96 (13)
C2—C1—C6	117.67 (14)	C13—C14—C15	118.23 (13)
C3—C2—C1	120.89 (16)	N1—C15—C14	112.01 (10)
C3—C2—H2	119.6	N1—C15—H15A	109.2
C1—C2—H2	119.6	C14—C15—H15A	109.2
C2—C3—C4	121.11 (16)	N1—C15—H15B	109.2
C2—C3—H3	119.4	C14—C15—H15B	109.2
C4—C3—H3	119.4	H15A—C15—H15B	107.9
C5—C4—C3	120.06 (16)	N1—C16—C17	111.53 (12)
C5—C4—H4	120.0	N1—C16—H16A	109.3
C3—C4—H4	120.0	C17—C16—H16A	109.3
C4—C5—C6	121.12 (16)	N1—C16—H16B	109.3
C4—C5—H5	119.4	C17—C16—H16B	109.3
C6—C5—H5	119.4	H16A—C16—H16B	108.0
C7—C6—C5	121.68 (15)	C22—C17—C18	119.11 (13)
C7—C6—C1	119.21 (15)	C22—C17—C16	120.99 (12)
C5—C6—C1	119.11 (15)	C18—C17—C16	119.88 (13)
C8—C7—C6	122.54 (15)	C19—C18—C17	120.05 (14)
C8—C7—H7	118.7	C19—C18—H18	120.0
C6—C7—H7	118.7	C17—C18—H18	120.0
C7—C8—C9	122.15 (15)	C20—C19—C18	120.37 (14)
C7—C8—C13	119.11 (14)	C20—C19—H19	119.8
C9—C8—C13	118.74 (15)	C18—C19—H19	119.8
C10—C9—C8	121.28 (16)	C19—C20—C21	120.05 (14)
C10—C9—H9	119.4	C19—C20—H20	120.0
C8—C9—H9	119.4	C21—C20—H20	120.0
C9—C10—C11	120.10 (15)	C22—C21—C20	119.72 (15)
C9—C10—H10	119.9	C22—C21—H21	120.1
C11—C10—H10	119.9	C20—C21—H21	120.1
C12—C11—C10	120.87 (16)	C17—C22—C21	120.71 (13)
C12—C11—H11	119.6	C17—C22—H22	119.6
C10—C11—H11	119.6	C21—C22—H22	119.6
			
C14—C1—C2—C3	−177.07 (13)	C7—C8—C13—C12	178.28 (12)
C6—C1—C2—C3	1.8 (2)	C9—C8—C13—C12	−1.57 (19)
C1—C2—C3—C4	−0.1 (2)	C2—C1—C14—C13	−178.15 (12)
C2—C3—C4—C5	−0.9 (2)	C6—C1—C14—C13	3.01 (19)
C3—C4—C5—C6	0.2 (2)	C2—C1—C14—C15	4.2 (2)
C4—C5—C6—C7	−178.11 (14)	C6—C1—C14—C15	−174.63 (12)
C4—C5—C6—C1	1.5 (2)	C12—C13—C14—C1	179.28 (12)
C14—C1—C6—C7	−3.89 (19)	C8—C13—C14—C1	0.21 (19)
C2—C1—C6—C7	177.20 (12)	C12—C13—C14—C15	−3.02 (19)
C14—C1—C6—C5	176.47 (12)	C8—C13—C14—C15	177.92 (12)
C2—C1—C6—C5	−2.44 (19)	C16—N1—C15—C14	−170.73 (11)
C5—C6—C7—C8	−178.83 (13)	C1—C14—C15—N1	−106.13 (14)
C1—C6—C7—C8	1.5 (2)	C13—C14—C15—N1	76.17 (15)
C6—C7—C8—C9	−178.44 (13)	C15—N1—C16—C17	52.24 (15)
C6—C7—C8—C13	1.7 (2)	N1—C16—C17—C22	81.05 (17)
C7—C8—C9—C10	−179.48 (14)	N1—C16—C17—C18	−97.18 (15)
C13—C8—C9—C10	0.4 (2)	C22—C17—C18—C19	−0.7 (2)
C8—C9—C10—C11	0.6 (2)	C16—C17—C18—C19	177.52 (14)
C9—C10—C11—C12	−0.4 (2)	C17—C18—C19—C20	0.6 (2)
C10—C11—C12—C13	−0.9 (2)	C18—C19—C20—C21	−0.1 (2)
C11—C12—C13—C14	−177.20 (13)	C19—C20—C21—C22	−0.2 (2)
C11—C12—C13—C8	1.9 (2)	C18—C17—C22—C21	0.5 (2)
C7—C8—C13—C14	−2.6 (2)	C16—C17—C22—C21	−177.76 (13)
C9—C8—C13—C14	177.55 (11)	C20—C21—C22—C17	0.0 (2)

### Hydrogen-bond geometry (Å, °)

**Table:**

*D*—H···*A*	*D*—H	H···*A*	*D*···*A*	*D*—H···*A*
N1—H1A···Cl1^i^	0.92 (1)	2.26 (1)	3.0963 (13)	152 (1)
N1—H1B···Cl1	0.92 (1)	2.17 (1)	3.0781 (16)	170 (1)
C16—H16A···Cl1^ii^	0.97	2.60	3.4824 (16)	151

Symmetry codes: (i) −*x*+1, −*y*+1, −*z*+1; (ii) −*x*, −*y*+1, −*z*+1.

## References

[bb1] Ashton, P. R., Ballardini, R., Balzani, V., Marcos, G.-L., Lawrence, S. M., Victoria, M.-D., Montalti, M., Piersanti, A., Prodi, L., Stoddart, J. F. & Williams, D. (1997). *J. Am. Chem. Soc.* **119**, 10641–10651.

[bb2] Nakazono, K., Kuwata, S. & Takata, T. (2008). *Tetrahedron Lett.* **49**, 2397–2400.

[bb3] Rigaku/MSC (2005). *CrystalClear* Rigaku/MSC Inc., The Woodlands, Texas, USA.

[bb4] Sheldrick, G. M. (2008). *Acta Cryst.* A**64**, 112–122. 10.1107/S0108767307043930 18156677

